# The impact of goat hair as a natural animal fiber on properties of the lightweight cement composite

**DOI:** 10.1038/s41598-025-91130-9

**Published:** 2025-02-26

**Authors:** Najmeh Hassas, Farzam Omidi Moaf, Marzena Kurpińska, Teresa Bardzińska-Bonenberg, Justyna Borucka, Hakim S. Abdelgader, Rohan Soman, Mikołaj Miśkiewicz

**Affiliations:** 1 Faculty of Architecture, Gdansk University of Technology, Gdańsk, Poland; 2 Faculty of Civil and Environmental Engineering, Gdansk University of Technology, Gdańsk, Poland; 3 Institute of Fluid-Flow Machinery Polish Academy of Sciences, Gdańsk, Poland; 4 Faculty of Architecture and Design, University of the Arts Poznan, Poznan, Poland; 5 Civil Engineering Department, Faculty of Engineering, University of Tripoli, Tripoli, Libya

**Keywords:** Natural fibre, Eco-friendly cement composites, Reinforcement composites, SEM, Civil engineering, Structural materials

## Abstract

The increasing demand for sustainable and eco-friendly construction materials has prompted the exploration of natural fibers as reinforcement in cement composites. This study investigates the potential of goat hair as a natural fiber reinforcement in lightweight cement composites to enhance mechanical properties and sustainability. The research evaluates goat-hair-reinforced composites’ flexural and compressive strengths at 7 and 28 days after mixing. The results show that the inclusion of goat hair at a rate of 0.4% of cement, as a reinforcing material, leads to a significant increase in flexural and compressive strength. Specifically, flexural strength increased by 2.5% and 21.8%, while compressive strength improved by 5.5% and 21.5% at water-to-cement ratios of 0.4 and 0.5, respectively, compared to the control mixture. The findings demonstrate the effectiveness of goat hair in improving mechanical performance while reducing the environmental footprint of construction materials. This study highlights the need for further exploration of natural fibers in sustainable construction practices, focusing on optimizing mechanical performance and eco-friendly materials for broader applications.

## Introduction

The integration of sustainable and innovative materials into the construction industry represents a significant leap towards eco-friendly and efficient building practices. This trend not only addresses the growing environmental concerns associated with conventional construction materials but also explores the untapped potential of natural and by-product resources. Several types of research were conducted on the use of different fibres as reinforcement in concrete^1–5^.

It has been proven that the modification of the cement matrix by adding dispersed fibers reduces shrinkage and it has a positive impact on the cracking mechanics of mortars and concrete^6–10^. Modification of physical and mechanical properties of mortars and concretes by natural fibres makes the structure more resistant to load and shrinkage of elements^10–15^. Natural fibers, such as sisal, coir, and flax, not only enhance the structural integrity of cement composites but also promote environmental sustainability by reducing reliance on synthetic reinforcements^16–20^. The inclusion of natural fibers has been found to improve both the physical and mechanical properties of cementitious materials, making them more resistant to loads and shrinkage-induced deformations. For instance, sisal fibers have demonstrated improvements in compressive strength by up to 6%, tensile strength by 14%, and flexural strength by 11% in lightweight concrete applications^21,22^. Similarly, basalt fibers and hypo sludge have been effectively used to enhance post-cracking behavior and overall durability, further underscoring the potential of natural and recycled materials in sustainable construction practices^23^.

This article delves into the pioneering research on the use of goat hair and GGA in lightweight concrete. By examining the unique properties and potential applications of these materials, this study aims to contribute to the evolution of construction methodologies, emphasizing sustainability, performance, and innovation.

Goat hair, a material with a storied history in the construction of the Black Tents of Iranian nomads, offers remarkable durability and weather resistance. These tents, emblematic of a sustainable and nomadic lifestyle, have withstood harsh environmental conditions for centuries, a testament to the material’s resilience. This not only reinvigorates a traditional material for contemporary use but also aligns with the increasing demand for construction practices that prioritize environmental sustainability and material efficiency^24,25^.

Parallel to the exploration of goat hair, the study sheds light on the innovative application of GGA) in producing lightweight concrete. GGA’s low density, coupled with its thermal insulation and fire-resistant properties, presents a compelling alternative to traditional aggregates. By reducing the overall weight of concrete, GGA contributes to a decrease in structural load demands, which is particularly advantageous for high-rise buildings and expansive structures. Furthermore, its utilization aligns with the ecological imperative of reducing the carbon footprint of construction materials, showcasing the feasibility of integrating eco-friendly by-products into the industry^26,27^.

The fusion of goat hair and GGA in lightweight concrete exemplifies a multifaceted approach to material innovation. This combination not only leverages the individual strengths of each material—such as improved mechanical properties and environmental sustainability—but also signifies a step towards reconciling traditional practices with modern construction requirements. The study meticulously evaluates the interactions between these materials and the cement matrix, aiming to optimize the composite’s performance for practical applications. By doing so, it sets a precedent for the development of building materials that are not only cost-effective and durable but also environmentally responsible.

This research introduces a novel integration of goat hair, a natural animal fiber with proven durability and historical significance, into lightweight cement composites. While the use of plant-based and synthetic fibers in cement composites is well-documented, the application of goat hair remains largely unexplored. This study uniquely combines goat hair with GGA to enhance the mechanical and sustainability properties of lightweight composites. By identifying the optimal fiber-to-cement ratio (0.4%) and establishing a predictive relationship between tensile and compressive strengths, this research provides quantitative and practical insights for field applications. Furthermore, the dual focus on natural fibers and lightweight aggregates represents a sustainable advancement in material innovation, aligning with global efforts to reduce the carbon footprint of construction materials.

The implications of this research extend beyond the immediate advancements in building material technology. It represents a paradigm shift in the construction industry’s approach to sustainability, material sourcing, and innovation. As the world grapples with the dual challenges of environmental degradation and the need for durable infrastructure, the integration of natural fibers and innovative aggregates offers a promising path forward. Moreover, this study contributes to the broader discourse on sustainable construction practices, encouraging further research and experimentation with other natural and recycled materials.

## Materials and methods

The selection of lightweight mortar as the focal point of this research is predicated on several compelling considerations. Primarily, lightweight mortar offers significant advantages as an insulating material, which, when coupled with the simplicity and directness of mortar testing methods, underscores its suitability as a medium for investigation. Consequently, this choice facilitates a nuanced exploration of concrete’s performance characteristics by examining the effects of incorporating goat hair fibers into the mortar. This study is dedicated to a meticulous quantitative analysis of the tensile and compressive strengths of concrete, reinforced with this particular type of animal fiber. Such an inquiry not only broadens the understanding of lightweight mortar’s utility but also contributes to the broader discourse on enhancing construction materials with sustainable and innovative reinforcements.

### Materials

**Fig. 1:**
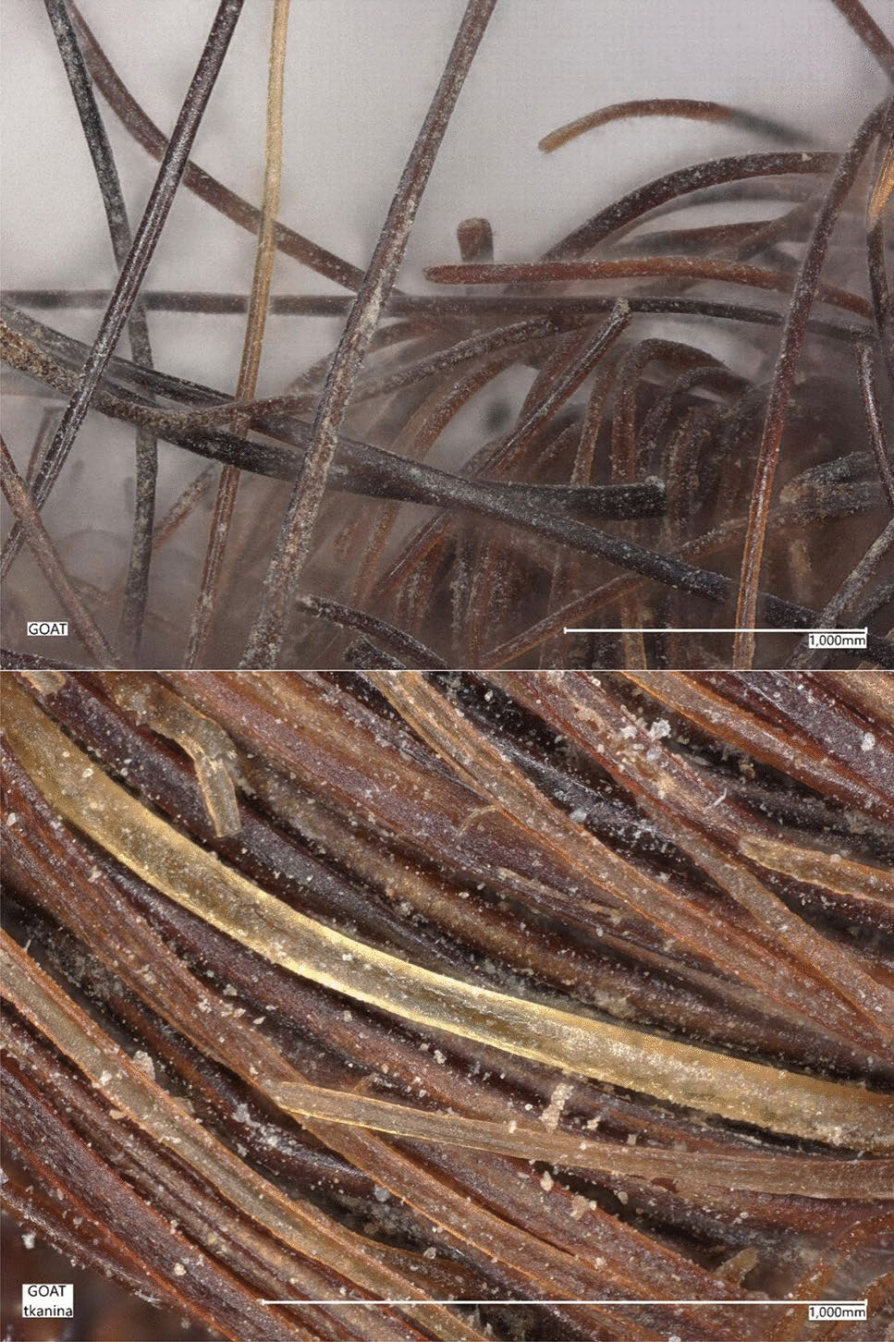
Goat hair Fibres used in research (100 × zoom and 200 × zoom).

The experimental phase involved rigorous evaluations of non-standard lightweight composites, which were ingeniously modified by incorporating goat hair fibers. Figure 1 shows a visual image of the goat hair fibers used in this study, which are obtained from the goat hair of the Qashqai tribe nomads around Shiraz, Iran. The distinctive properties of the goat fibers are systematically cataloged in Table 1, with a uniform fiber length of 19 mm being a standard parameter across all fiber types examined^14^.

**Table 1:** Properties of goat hair fibres used in research^14^.

Property	Unit	Value
Length (l)	(mm)	19
Diameter (d)	(mm)	0.003
Slenderness (l/d)	(–)	6333
Number of fibres	(per 1 kg) (× 10^3^)	7,445,845
Total length	(m/kg) (× 103)	141,471
Total length of reinforcement (40 × 40 × 160 mm)	(m)	65,076.7
Density	(g/cm^3^)	1.5–1.6
Young’s modulus	(GPa)	5.5–12.6
Tensile strength	(MPa)	287–800
Elongation	(%)	3–10
Cellulose	(%)	80–94
Hemicellulose	(%)	–
Lignin	(%)	–
Natural moisture	(%)	0
Moisture absorption after 24h	(%)	25–50

The intricate morphology of these fibers was meticulously analyzed through the use of a high-resolution digital microscope, model VHX-7000N, manufactured by KEYENCE in Tokyo, Japan. This phase of the research was strategically designed to elucidate the detailed surface structure as well as the cross-sectional characteristics of the fibers, thereby providing invaluable insights into their potential implications for the composite material’s overall performance.

The selection of CEM II/B-V cement for this study was primarily motivated by its comparatively lower clinker content, approximately 65%, positioning it as a more environmentally sustainable option. This attribute significantly reduces its carbon footprint to 704 kg per tonne of cement, presenting a marked improvement over the CEM I cement variant, which, with a clinker content of approximately 95%, is associated with CO_2_ emissions of around 889 kg per tonne of cement. The economic viability of CEM II/B-V cement renders it a preferred choice in the fabrication of fiber-reinforced concrete composites.

Furthermore, empirical evidence has consistently demonstrated that this type of cement exhibits superior performance in composites reinforced with natural fibers. This enhanced compatibility and performance underscore the potential of CEM II/B-V cement not only as a sustainable building material but also as a catalyst for improving the structural integrity and environmental footprint of cement-based composites. The chemical composition and physical properties of CEM II/B-V 42.5R are shown in Table 2.

**Table 2:** Chemical composition and physical properties of CEM II/B-V 42.5R.

Setting start time (min)	Setting end time (min)	Compressive strength (MPa)		Blaine fineness (cm^2^/g)	Loss on ignition (%)	Water demand (%)			
		2 day	28 day						
200	255	25.3	48.1	4414	3.9	28.0			
Content (%)									
SiO_2_	Al_2_O_3_	Fe_2_O_3_	CaO	MgO	SO_3_	Na_2_O	K_2_O	TiO_2_	Cl^(-)^
25.2	8.9	3.4	54.5	1.3	2.5	0.21	0.66	0.15	0.06

**Fig. 2:**
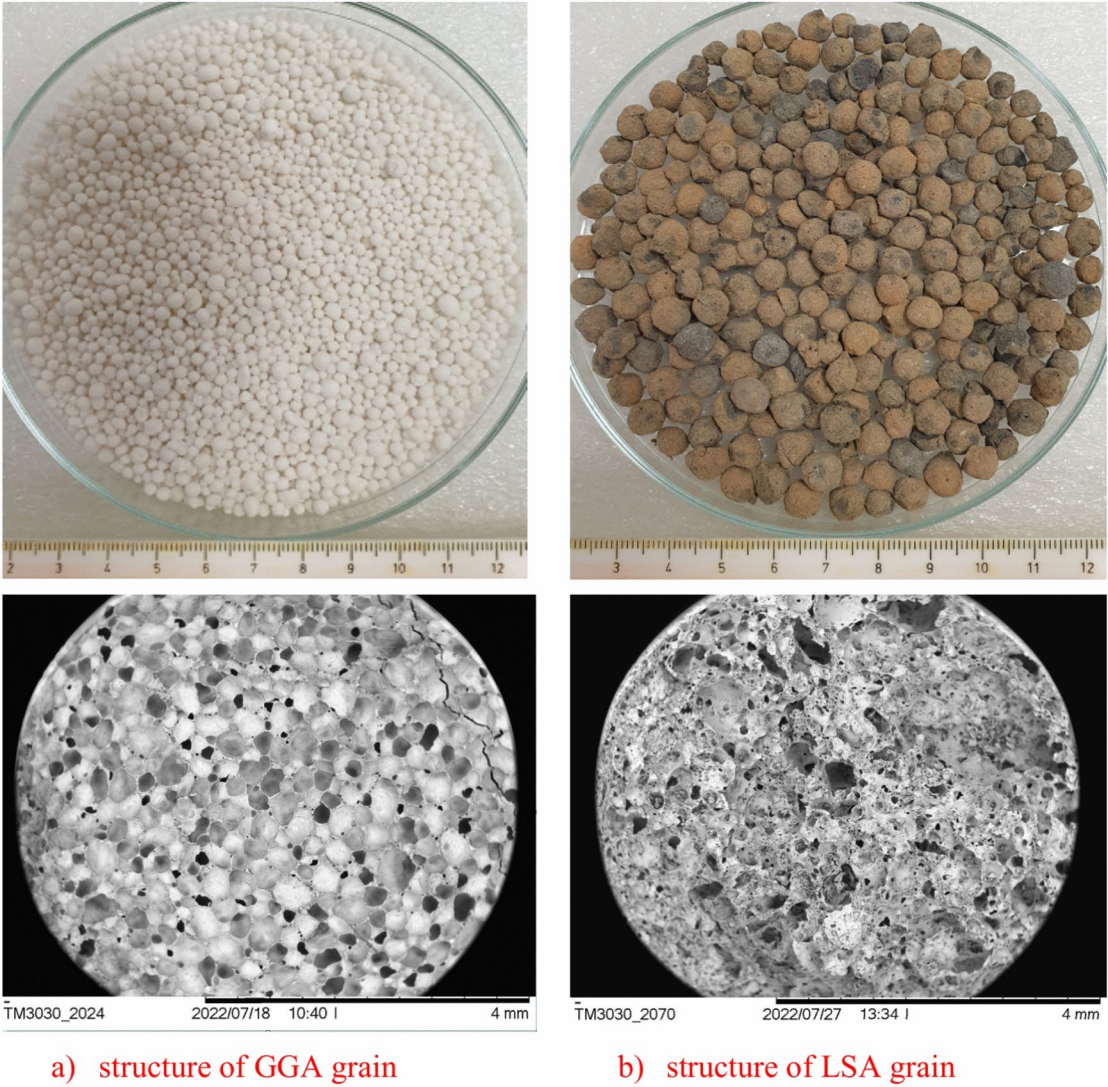
The lightweight aggregates.

**Fig. 3:**
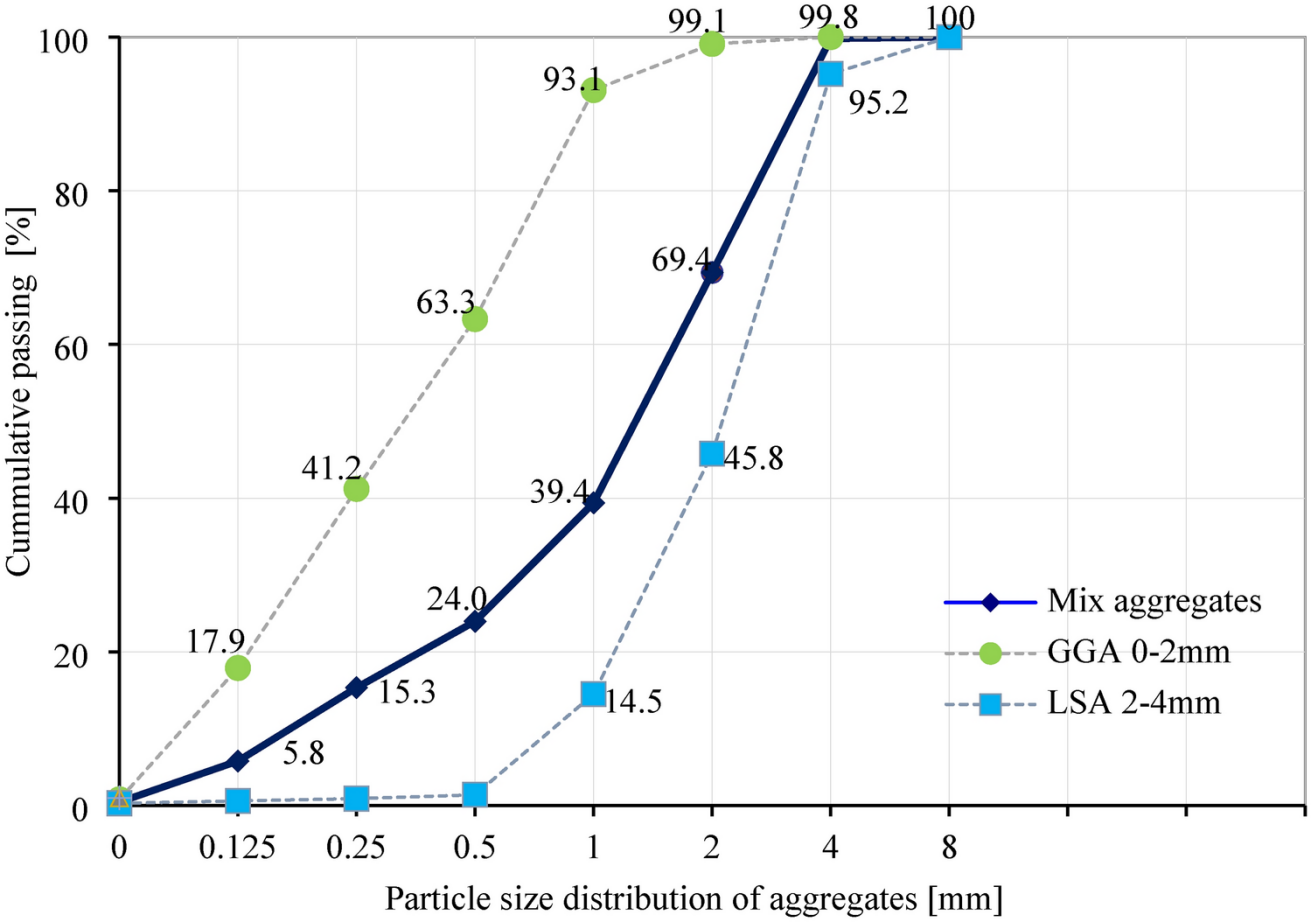
The lightweight aggregate screening curves.

As an aggregate was used as GGA with a grain diameter of 0.25–2 mm (Granulated Glass Aggregate, PORAVER, Germany) LSA with a grain diameter of 2–4 mm (Lightweight Sintered Ash, Białystok, Poland) (Fig. 2). The chemical composition of aggregates is presented in Table 3, and the physical and mechanical properties of are illustrated in Table 4. The grading details and shape of lightweight aggregates and mix aggregate are shown in Fig. 3.

**Table 3:** The chemical composition of lightweight aggregate.

	SiO_2_	Al_2_O_3_	Fe_2_O_3_	CaO	MgO	SO_3_	Na_2_O	K_2_O	Loss of roasting
Content (%)									
GGA	66.21	0.63	0.00	14.11	2.58	0.30	12.23	0.52	3.42
LSA	52.82	24.28	7.50	4.49	3.18	0.43	0.00	0.20	7.10

**Table 4:** The physical and mechanical properties of lightweight aggregate.

Particle size (mm)	Bulk density (kg/m^3^)	Water absorption (%)	Porosity (open/close) (%)	Thermal conductivity (W/m K)
GGA (2 mm)	360	15.8	16/54	0.065
LSA (4 mm)	1350	16.7	11/37	0.850

Clean water from municipal water supplies was used for the mix.

The Power Flow plasticizing admixture was used in the tests. The MC-PowerFlow 1106 admixture is based on the latest polycarboxylate technology. Thanks to accelerated adsorption on the binder grains, its immediate effect occurs. The recommended dosage is from 0.2 to 5% of the cement mass.

Six blends of lightweight composites modified with goat hair fiber were tested. The ratios of goat hair to cement were chosen as 0. 0.4% and 0.8% respectively. In addition the water/cement (w/c) ratios of 0.4 and 0.5 were selected based on their practical relevance in lightweight cement composites. A lower w/c ratio such as 0.4 typically enhances the density of the cement matrix by reducing porosity resulting in improved mechanical properties. In contrast a higher ratio like 0.5 ensures better workability which is particularly critical in mixes containing fibrous reinforcements as it facilitates uniform fiber dispersion and reduces the risk of clumping. These two values were chosen to represent a balance between achieving high mechanical performance and maintaining sufficient workability reflecting the practical constraints often encountered in construction applications. The composition of the mixtures is presented in Table 5.

**Table 5:** Mix design of composites for 1 kg/m^3^.

Materials	Density	w/c = 0.4			w/c = 0.5		
		0400	042G	044G	0500	052G	054G
		Unit (kg/m^3^)					
CEM II/B-V 42.5N	2.95	470			450		
Water (eff)	1.00	188			225		
LWA							
GGA 0–2	0.55	183			175		
LSA 2–4	1.35	427			407		
PowerFlow 1106 FM 1% c.c	1.07	4.7			4.5		
Goat hair (G)	0.82	0	2	4	0	2	4

### Methods

**Fig. 4:**
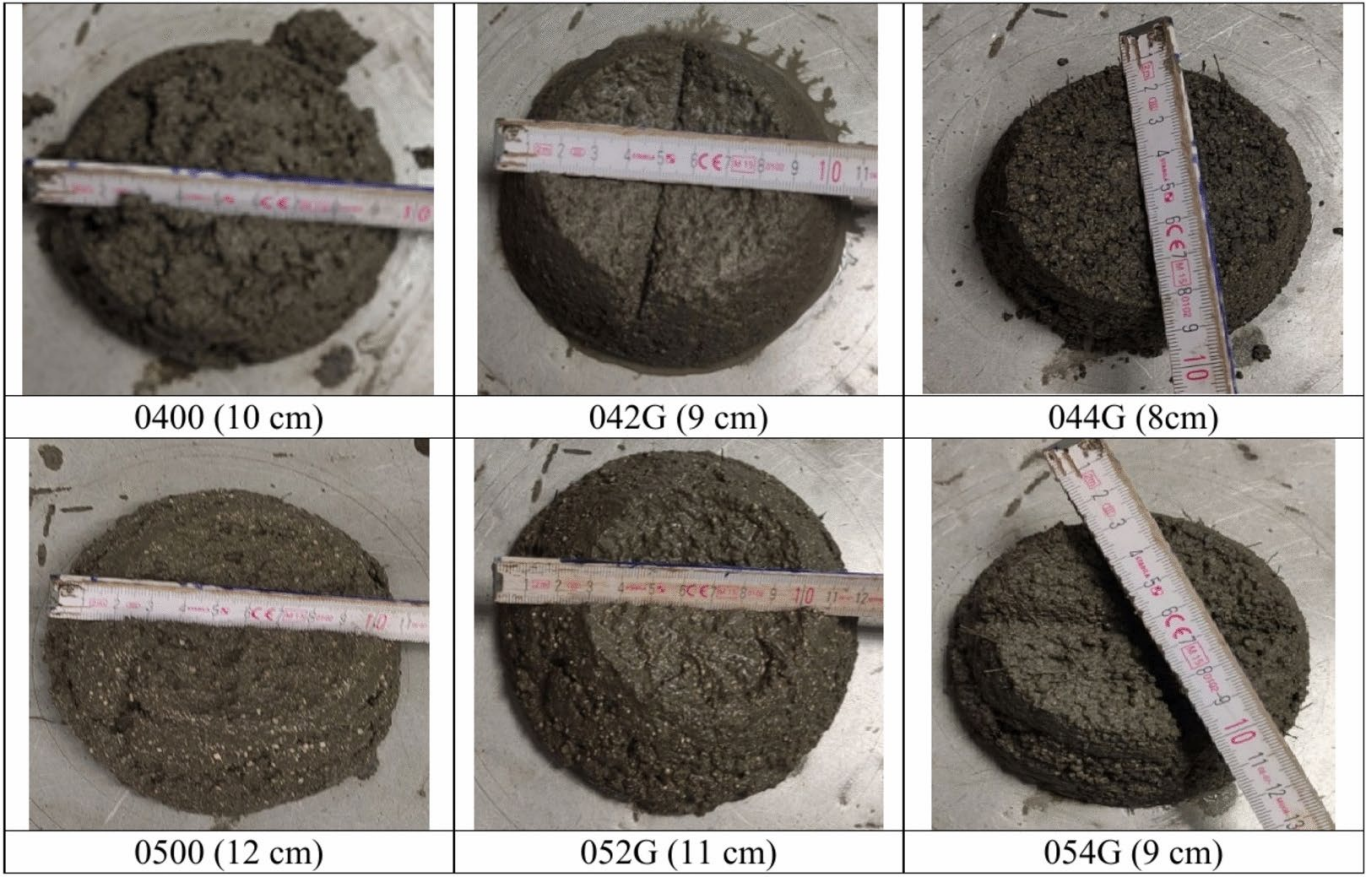
Consistency testing.

The lightweight mix was prepared in an appropriate mixer (Automatic Mortar Mixers. AUTOMIX. Controls). In the first stage cement and water were dosed and blended then the fibers were added together with fine lightweight aggregate. All ingredients were mixed for 2 min. Next the lightweight fiber-composite was filled into three-part metal molds in two layers. A total of 36 composite samples (6 samples of each type) in size of 4 × 4 × 16 cm were prepared for the flexural strength test and after that 72 samples (12 samples of each type) in size of 4 × 4 cm were used for compressive strength testing. Composite consistency tests were carried out on a shock table designed to test the mortars according to EN 196-1^28^. The measure of consistency is the measurement of two perpendicular diameters in (mm) of the mixture flow of the mix (Fig. 4).

In order to assess the mechanical properties of the hardened lightweight fibre composites the research on the flexural strength and compressive strength was carried out. These tests were conducted on the samples with the dimensions of 4 × 4 × 16 cm with the use of an endurance machine manufactured by CONTROLS ADVANTEST 9 (Controls. Italy) with a maximum pressure of 300 kN.

**Fig. 5:**
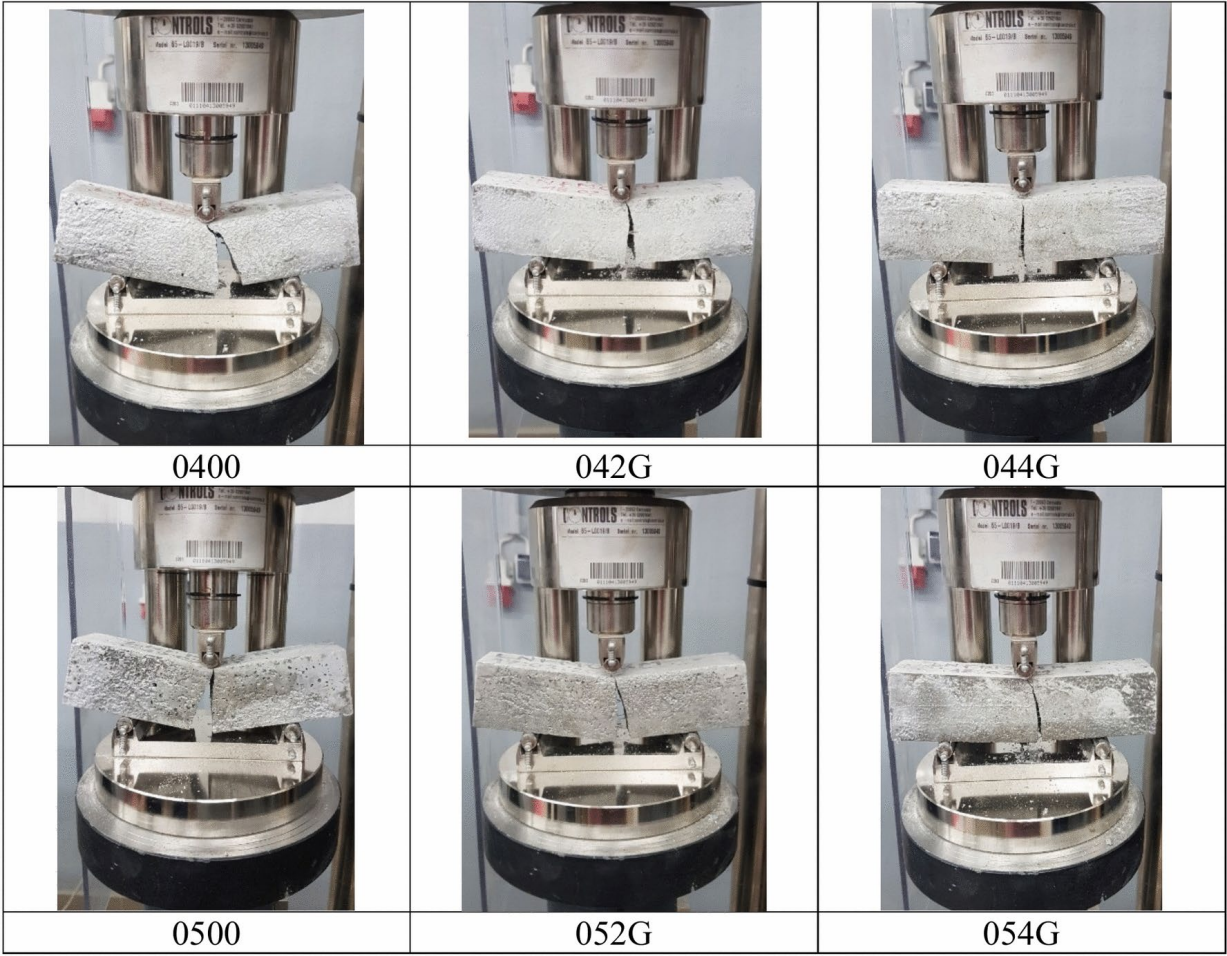
Scheme of the flexural strength test.

In the first step was flexural strength test was made on samples without notch in a 3-axis bending scheme. The bending force was applied at the centre of the sample span. The distance between the supports was 100 mm (Fig. 5).

The flexural strength of the composite was calculated according to the formula (1):

1 $$\sigma = \frac{2Fl}{{3hd^{2} }}$$

where: $\sigma$—flexural strength [MPa]. $F$—load (force) at the fracture point [N]. $l$-the length of the support span [mm. $h$—width of samples [mm]. $d$—thickness of samples [mm].

Evaluation of the flexural strength of a group of three prisms is used as the test result.

**Fig. 6:**
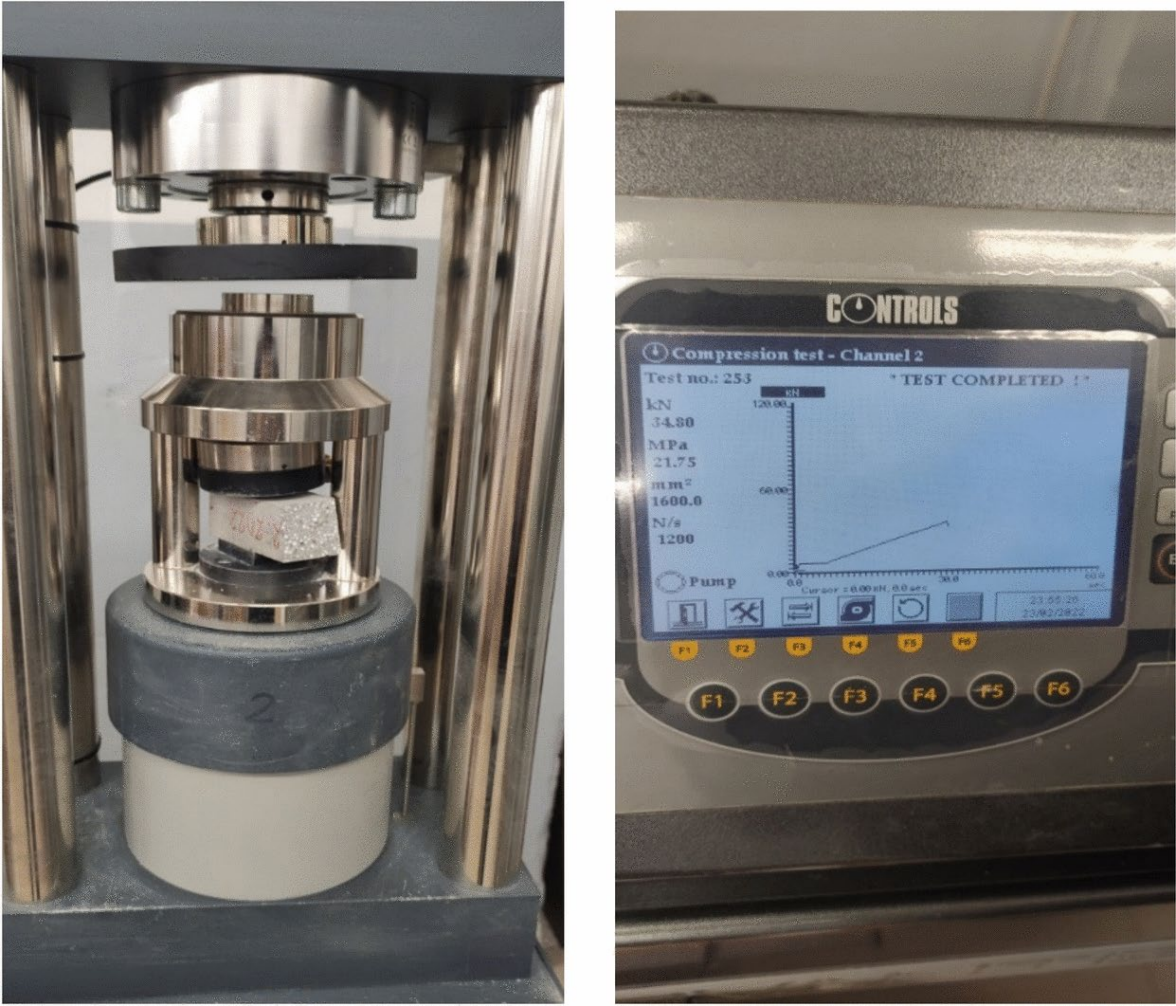
Compressive strength test.

At the compressive strength test the compression surface of the specimen was 40 × 40 mm (Fig. 6). Both tests were carried out in accordance with standard EN 196-1.

The compressive strength of concrete is calculated according to the formula (2):

2 $$\sigma = \frac{F}{{A_{0} }}$$

where $\sigma$—compressive strength [MPa]. $F$—load applied [N]. ${A}_{0}$—area [mm^2^].

## Results and discussion

Assigning mechanical properties of fibre-reinforced composite particular emphasis was placed on the determination of the flexural strength of the composite. This parameter was appointed by the 3-point test. Figure 6 shows the flexural strength of different samples on the 7th and 28th days. For the 7-day test one sample of each type was used and for the 28-day test. 5 samples of each type were used and the standard deviation of each group is shown in Fig. 6.

**Fig. 7:**
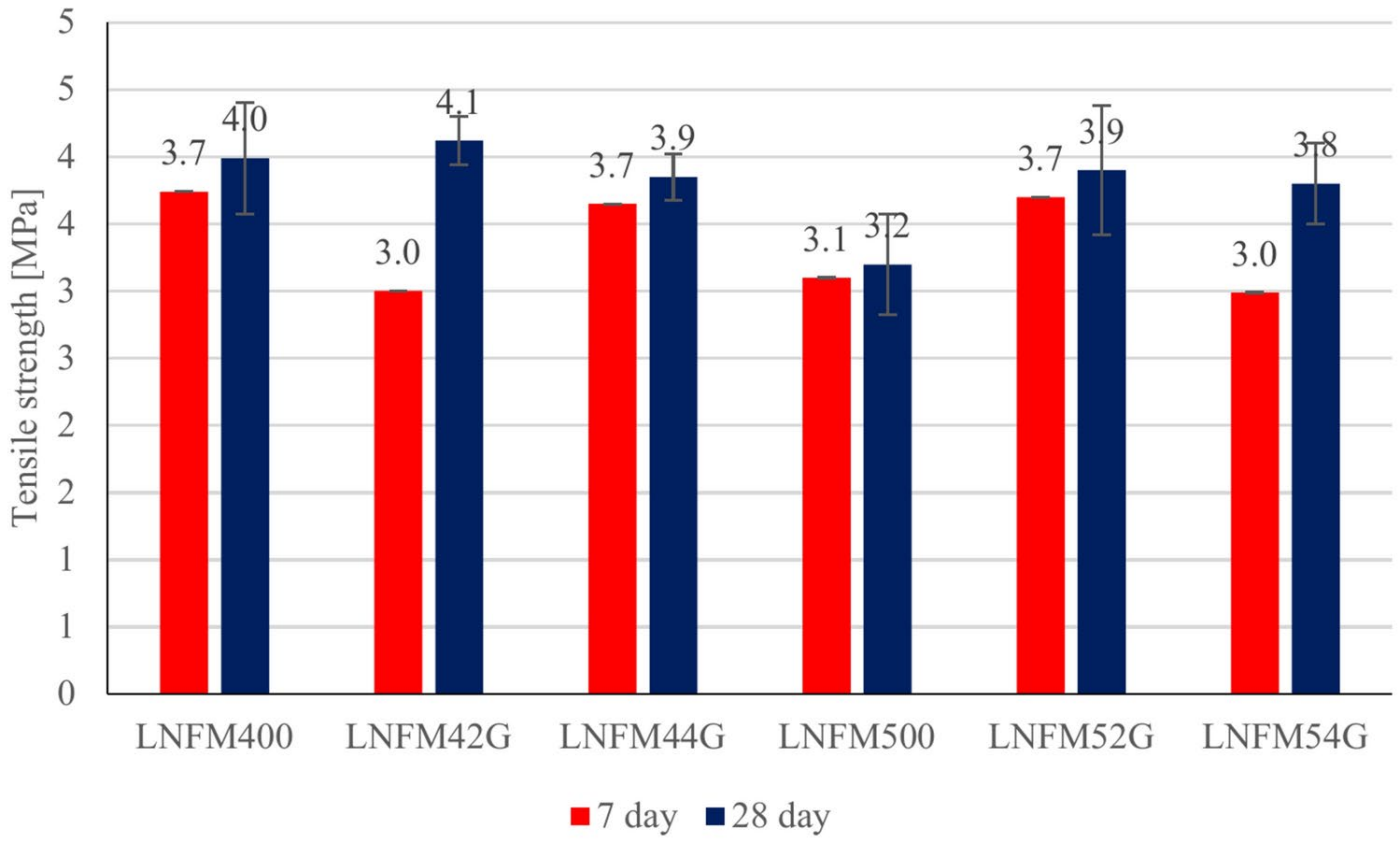
Flexural strength test results.

The flexural strength results for the different samples tested on the 7th and 28th days are presented in Fig. 7. For the 7-day test the flexural strength values ranged from 3.0 to 3.7 MPa across the different mixes. Notably the LNFM52G sample (containing 0.8% goat hair) achieved the highest flexural strength of 3.9 MPa at 28 days demonstrating a 21.8% improvement compared to the control mix’s 3.2 MPa. But the highest flexural strength is 4.1 MPa for LNFM42G which contains 0.4% goat hairs.

The inclusion of goat hair fibres positively influenced flexural performance over time as evidenced by the marked improvements between the 7th and 28th days. These results align with previous studies such as Prakash et al. (2020) which reported an 11% improvement in flexural strength using sisal fibre in lightweight concrete^22^. While synthetic fibers such as nylon have achieved up to 52.8% improvements the environmental benefits and competitive performance of goat hair underscore its potential as a sustainable alternative^10^.

**Fig. 8:**
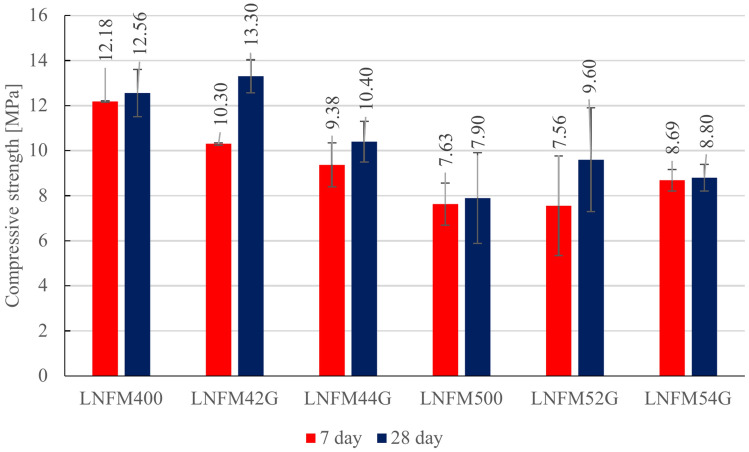
Compressive strength test results.

Figure 8 illustrates the compressive strength results which followed a similar trend to the flexural strength findings. For the 7-day test. 2 samples of each type were used and for the 28-day test. 10 samples of each type were used and the standard deviation of each group is shown in Fig. 8.

The compressive strength results demonstrate a consistent trend of improvement over time for all samples. On the 7th day compressive strength ranged from 7.63 to 12.18 MPa with LNFM400 and LNFM42G exhibiting the highest values. By the 28th day compressive strength ranged from 7.96 to 13.3 MPa with LNFM42G (0.4% goat hair) achieving the highest strength. The 21.5% increase occurred in compressive strength LNFM52G compared to the control mix (LNFM500) which reached only 11.5 MPa at 28 days.

These findings align with results from Sekar et al. (2023) where basalt fibers and hypo sludge achieved a 17.08% improvement in compressive strength^23^. Although synthetic fibers such as nylon achieve slightly higher compressive strength gains goat hair offers comparable performance while aligning with sustainability goals^22^.

These results underscore the effectiveness of goat hair as a reinforcing agent in lightweight cement composites improving both the immediate and long-term mechanical properties of the material.

**Fig. 9:**
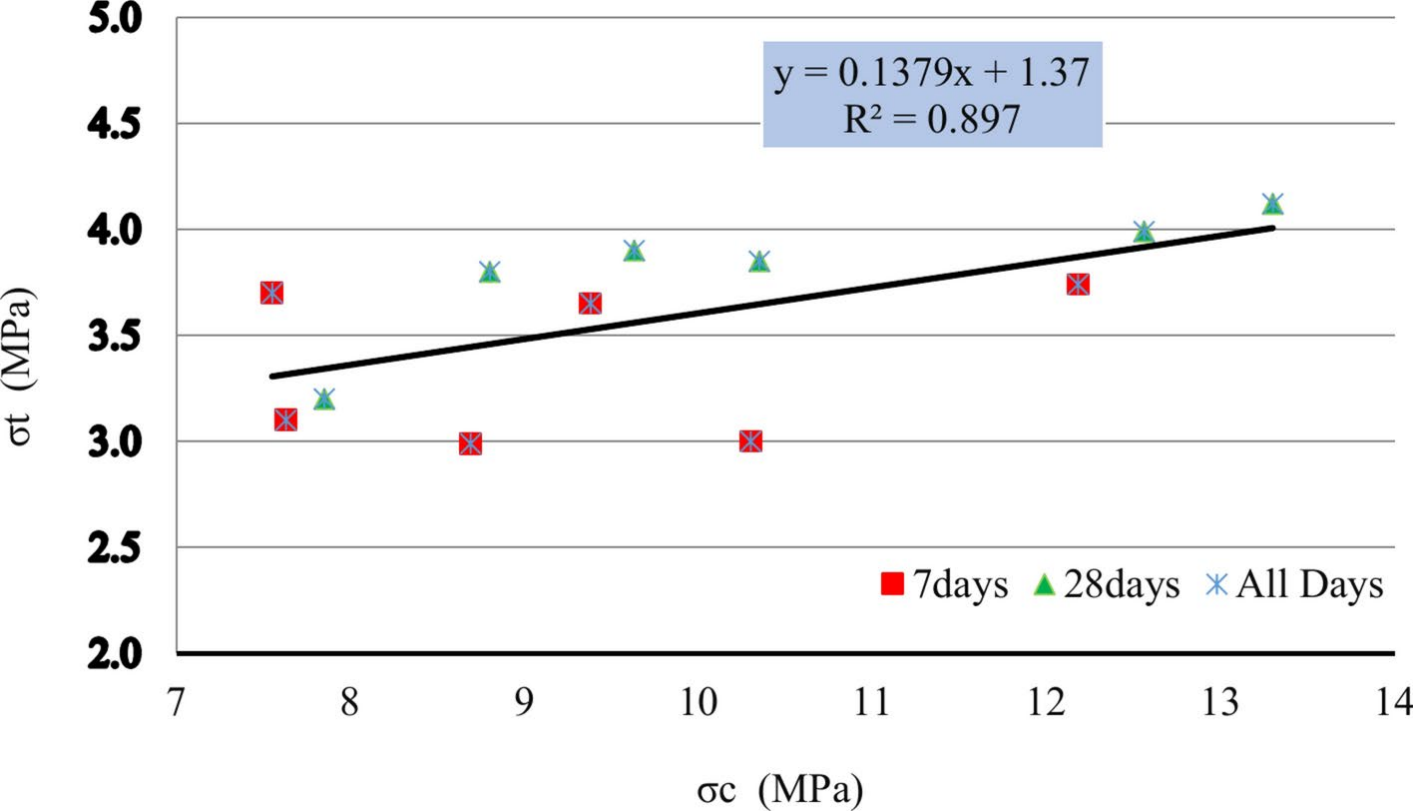
Compressive strength test results.

By analyzing the results of tensile strength and compressive strength a strong linear relationship between these two parameters was calculated which can be seen in formula 3 and Fig. 9.

3 $${\text{All}}\;{\text{days}}:\sigma_{t} = 0.1379\sigma_{c} + 1.37\;with\;R^{2} = 0.897$$

Equation can be used to predict the tensile strength of the mortar based on its compressive strength. This formula can be useful in practical applications where estimating one parameter based on the other is necessary making it easier to determine the tensile strength from known compressive strength values for this type of mortar.

## Conclusions

The research provided clear evidence of the positive impact of goat hair fiber on the mechanical properties of lightweight cement composites. During the study it was observed that the inclusion of goat hair not only enhanced the flexural and compressive strengths but also contributed to a more sustainable construction material profile.

Goat hair fibers proved to be particularly effective in increasing the strength parameters over time which is crucial for their application in real-world construction scenarios where durability and longevity are paramount. The increase in both flexural and compressive strengths over the 28-day period highlights the potential of these natural fibers to improve the performance of cement composites significantly.

The results showed that the cement mortar with 0.4% weight ratio of goat hair to cement has the best tensile and compressive performance.

The inclusion of goat hair as a reinforcing material at 0.4% of cement weight significantly enhances the mechanical properties of concrete. At a water-to-cement ratio (w/c) of 0.4 flexural strength increased by 2.5% and compressive strength improved by 5.5% while at a w/c ratio of 0.5 flexural strength rose by 21.8% and compressive strength by 21.5% compared to the control mixture. These findings highlight the effectiveness of goat hair in improving both flexural and compressive strength with more pronounced benefits at higher water-to-cement ratios.

Also the formula for calculating the tensile strength was calculated from the compressive strength for this type of mortar the strong linear relationship indicated by the high R^2^ value and the consistency of data points over different testing periods suggests that the provided formula is reliable for calculating the tensile strength from compressive strength for this type of mortar.

In conclusion the use of goat hair in cement composites offers a promising avenue for enhancing the mechanical properties of building materials while also contributing to environmental sustainability. The research supports the continued exploration and optimization of natural fibre composites in the construction industry advocating for the broader adoption of eco-friendly and durable building materials.

## Future research

Future research should aim to evaluate a wider range of fiber content levels to further optimize the mechanical performance and workability of lightweight cement composites. Testing intermediate fiber content ratios such as 0.5% and 0.6% as well as higher levels will provide valuable insights into the maximum reinforcement potential of goat hair while maintaining mix consistency and acceptable workability. These experiments will help establish the optimal fiber dosage for achieving superior mechanical performance without compromising practicality.

In addition a broader investigation of water-to-cement (w/c) ratios is necessary to deepen the understanding of their impact on the mechanical and rheological properties of goat-hair-reinforced composites. Testing intermediate values such as 0.35. 0.45 and 0.55 can help identify the ideal balance between workability fiber dispersion and mechanical strength which is crucial for adapting these materials to diverse construction applications.

Further research should also incorporate detailed microstructural analysis of goat-hair-reinforced composites using Scanning Electron Microscopy (SEM). This advanced technique can provide a visual representation of fiber-matrix interactions shedding light on bonding quality potential void formation failure mechanisms and the role of fiber morphology in enhancing mechanical properties. Understanding these microstructural behaviors will contribute to the design and optimization of high-performance durable and sustainable fiber-reinforced composites.

By addressing these aspects future studies will pave the way for broader adoption and improved performance of goat-hair-reinforced lightweight cement composites in modern construction.

## Data Availability

The datasets used and/or analysed during the current study available from the corresponding author on reasonable request.
